# Selenium in Germinated Chickpea (*Cicer arietinum* L.) Increases the Stability of Its Oil Fraction

**DOI:** 10.3390/plants8050113

**Published:** 2019-04-27

**Authors:** Daniela Guardado-Félix, Sergio O. Serna-Saldivar, Janet A. Gutiérrez-Uribe, Cristina Chuck-Hernández

**Affiliations:** 1 Tecnologico de Monterrey, Centro de Biotecnología FEMSA, Escuela de Ingeniería y Ciencias, Campus Monterrey, Eugenio Garza Sada 2501, Monterrey 64849, Mexico; daniela.guardado@tec.mx (D.G.-F.); sserna@tec.mx (S.O.S.-S.); 2 Programa Regional de Posgrado en Biotecnología, Facultad de Ciencias Químico-Biológicas, Universidad Autónoma de Sinaloa, FCQB-UAS, AP 1354, Culiacán, Sinaloa 80000, Mexico; 3 Tecnologico de Monterrey, Escuela de Ingeniería y Ciencias, Campus Puebla, Vía Atlixcáyotl 2301, Puebla 72453, Mexico; jagu@tec.mx

**Keywords:** cellular antioxidant activity, chickpea oil, germination, oxidative stability, selenium

## Abstract

Selenium is an essential mineral in human nutrition. In order to assess its effect on the stability of chickpea oil, seeds were germinated and tested with different amounts of sodium selenite (0.0, 0.5, 1.0 and 2.0 mg/100g seeds) for four days. Oil was extracted from sprouted chickpea and its physical properties, fatty acid profile (FAME), oxidative stability index (OSI), lipase and lipoxygenase (LOX) activities, cellular antioxidant activity (CAA), and phenolics and carotenoids were assessed and compared to chickpea seed oil. The amount of chickpea oil and its acid value (AV) increased during germination. The OSI increased by 28%, 46% and 14% for 0.5, 1.0 and 2.0 mg/100g compared with non-selenium treated sprouts. Phenolics increased up to 36% and carotenoids reduced by half in germinated sprouts with and without selenium compared to seeds. Carotenoids increased by 16% in sprouts treated with 1.0 mg/100 g selenium compared to their counterparts without selenium. FAME was not affected by treatments but samples with the highest selenium concentration increased lipase activity by 19% and decreased lipoxygenase activity by 55% compared with untreated sprouts. The CAA of oils increased by 43% to 66% in all germinated treatments compared with seeds. Results suggest that Se-enriched chickpea sprouts could represent an excellent source of oil with a high OSI and CAA, associated with a reduction in LOX activity and an increase in phenolics, respectively.

## 1. Introduction

Selenium (Se) is an essential mineral for humans and its intake is limited by the amounts that crops absorb from the soil. Cereals and legumes grown in Se-poor soils are the most common cause of its deficiency in some parts of the world [1]. We have found that sodium selenite (Na_2_SeO_3_) added during chickpea seed germination (up to 2 mg/100 g seeds) increases isoflavonoids and antioxidant activity in the sprouts and enhances glutathione peroxidase (GPx) and thioredoxin reductase (TrxR) activities [2]. GPx exerts chemopreventive effects because it reduces lipid hydroperoxide concentration and restricts lipid oxidation in cells as an energy-depriving mechanism [3]. The oxidative protection of selenium has been widely studied from the food functional point of view [2,4,5,6,7]. However, its protective effect over the oil fraction of commercial oilseeds and pulses has not been previously reported to the best of our knowledge, except by the recent work [8] on olive oil, when selenium was applied with foliar fertilization. The oxidative protection of selenium in vegetable oils has industrial and commercial interest because of its beneficial impact on food preservation.

Chickpea oil is an adequate model for oxidation study due to is unique fatty acid profile, which is high in polyunsaturated acids (66%), and rich in linoleic (18:2 Δ 9, 12) and monounsaturated oleic (18:1 Δ 9) acids, accounting for 51% and 32%, respectively of the total fatty acids. These percentages are higher when compared to lentils, peas and beans [9]. PUFAs are highly unstable and prone to deterioration when exposed to external factors such as air, ultraviolet light, temperature and internal factors, such as reactive oxygen species (ROS) and enzymatic activity. Besides the high percentage of PUFAs in chickpea, the high enzymatic activity (lipase and lipoxygenase (LOX)) contributes to lipid deterioration through hydrolysis and oxidation of fatty acids, being thus determinants in the nutritional quality of chickpea oil. Furthermore, chickpea composition changes due to the physiological events during germination. This process modifies the seed nutrient profile in preparation for growth and development of the embryo in a new plant. Among the changes is hormone production (gibberellins (GAs)), which in turn increases the activity of enzymes such as lipases. Lipases are responsible for hydrolysis of triglycerides to produce fatty acids and provide a source of energy and nutrients for the new plant [10]. The positive effect of selenium on mineral stress during germination over the increase in the activity of antioxidative enzymes has only been reported by [11], who researched the effect of cadmium over *Brassica napus* seedlings. Chickpea’s oil oxidative stability during germination with selenium stress has not been studied. The main objective of this research was to assess oxidative stability, total phenolics, carotenoids, lipase and LOX activities as well as antioxidant activity of Kabuli chickpea (*Cicer arietinum* L.) oil extracted from sprouts with different amounts of selenium. This is a different approach in the study of selenization to produce functional foods, because the interest is not the final product, but a mechanism aimed to improve oil stability during germination.

## 2. Results and Discussion

### 2.1. Physical and Chemical Properties of Oils Obtained from Chickpea Seeds and Seeds Germinated with Selenium

The effect of selenium in the physical and chemical properties of oil after four days of germination is shown in Table 1. Oil content in chickpeas is one of the highest among pulses. For instance, the average oil content of chickpeas (6.0%) is significantly higher when compared to common beans (*Phaseolus vulgaris* L.) and pigeon peas (*Cajanus cajan* L.) which contain 0.2 and 1.6% oil, respectively. In [12], the oil content in different chickpea cultivars was found to be in a range from 2.70 to 6.48%. Data depicted in Table 1 shows the crude fat content was 5.5% for the regular chickpea (without germination, nor selenization), which is within the range reported by [12]. The oil content increased significantly from 5.5 to 6.9% after four days of germination (Table 1), depicting the influence of sprouting in this chemical trait. In [13], germinated chickpeas started with 3.85% crude fat and finished after three days germination with 4.46%. In [14], an increase of crude fat after four days germination (5.0 to 7.7%) was also observed. The significant change could be associated with the common dry matter losses incurred during germination, especially from the cotyledons rich in protein and starch [15].

The germination process did not affect the oil refractive index (RI) (Table 1) as reported previously by [16]. RI is the ratio of the velocity of light in a vacuum to its velocity in a specified medium, in this case, oil. In oils, the RI can be used for evaluation of rancidity, and according to [17], values increase when the number of conjugated fatty acids also increases. Furthermore, RI is related to molecular weight and type of fatty acid, chain length and degree of unsaturation. As described in Table 1, there was an increasing trend in the RI for non-selenized sprouts. It has been reported that the RI of chickpea oil can vary between 1.48 and 1.49 [18], and for plant oils such as safflower, peanut, sesame and palm it was 1.477, 1.470, 1.473 and 1.465 respectively [19], close to the values reported herein.

The acid value (AV) in oil increases significantly during germination. The index is related to free fatty acid content and is an important indicator of vegetable oil quality. An increment in AV in oil indicates hydrolysis of triglycerides, a reaction that occurs during germination due to the augmentation in lipase A1 and A2 activities, enzymes synthesized to provide energy for seedling development [20]. In Table 1 it can be observed that the AV of the original chickpea seed was 5.0%, whereas in the sprouts without selenium, it was 12.4%. The highest level of AV of 15.6% was observed in the oil extracted from sprouts with 0.5 mg selenium.

As expected, the oxidative stability index (OSI) values of oils extracted from germinated chickpeas decreased due to the progressive catabolic process, but in the oil extracted from sprouts germinated with selenium (Table 1), a protective effect was observed. The OSI in oil from chickpea seeds was 31.8 h, more than twice that of the germinated sample (11.4 h). Among sprouted chickpeas, the 1.0 mg Na_2_SeO_3_ /100 g treatment depicted the highest OSI (16.7 h, Table 1). An interesting research showed that selenized brown rice sprouts, stored for nine months at different temperatures, maintained high quality and oxidative stability compared to their regular counterparts. The significant difference was attributed to the antioxidant activity of selenium and its inhibitory effect on lipid peroxidation [21]. Another study investigated the influence of selenium over olive oil, and the authors found that foliar application of this trace mineral exerted a positive effect on oil OSI and the effect was more intense when the olive trees were also subjected to drought stress [8]. According to these authors, water stress increased ROS production, which reduced oil stability. It is known that selenium is involved in stress tolerance through ROS detoxification, and helps to maintain the membrane integrity of the oil bodies and the stability of proteins and enzymes. In the case of mineral stress, as reported by [11], the increase of ROS was also observed and when H_2_O_2_ accumulated, the production of secondary metabolites increased. As shown in Table 1, germination decreased OSI as expected, because of the hydrolytic activity of lipases synthesized to yield energy for seedling development. The OSI of oils extracted from selenized sprouts compared with non-selenized counterparts had an increasing trend, implying a protective effect of selenium over the oil fraction. The observed effect is mainly attributed to the activity against lipid hydroperoxide production via GPx, decreasing the total lipid oxidation [3].

### 2.2. Fatty Acid Profiles (FAMEs) of Oils Obtained from Chickpea Seeds and Seeds Germinated with Selenium

Fatty acid profiles of oils extracted from chickpea seeds and sprouts showed significant amounts of unsaturated fatty acids, mainly linoleic acid, followed by oleic acid and lower concentrations of saturated palmitic and stearic acids (Table 2). These results are consistent with previous reports for chickpea oil [13]. No significant differences were found among treatments in terms of fatty acid profiles, similar to the results reported by [22] for olive oil, where authors did not find a different fatty acid profile between treatments for olives produced under water deprivation and foliar application of selenium.

### 2.3. Effect of Selenium on Lipase and LOX Activities of Chickpea Sprouts

**Figure 1:**
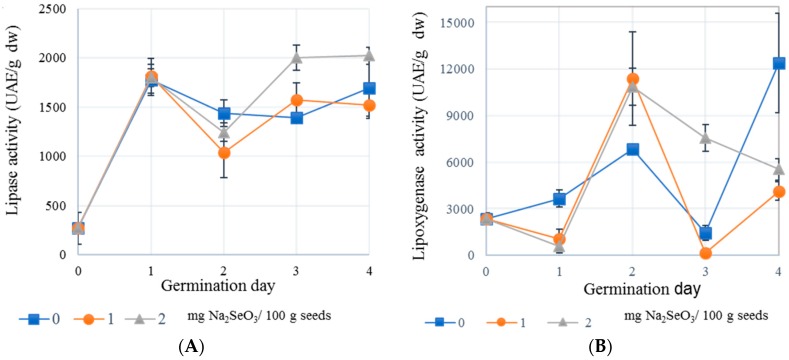
Effect of sodium selenite concentrations on lipase (**A**) and lipoxygenase (**B**) activities of chickpea sprouts germinated for four days. Values depicted are averages and bars represent standard deviations.

Lipases catalyze the hydrolysis of ester bonds in water insoluble lipid substrates. These enzymes are one of the first synthesized upon germination. The highest lipase activity was observed on the first day of germination in all treatments (Figure 1A), except in 2.0 mg/100g samples, where the highest activity was reached in the third and fourth day (43% and 19% more activity than sprouts germinated without selenium). A similar trend was previously reported by [23] for sesame seeds, where the highest lipolytic activity was reached at the fourth day of sprouting. The fact that lipase activity has a biphasic behavior (Figure 1A), has been also reported for coffee sprouts and it is associated with the activity of different iso enzymes (lipases I and II). The first being responsible for lipid metabolism in root and shoot growth and the second is active during germination, so the first activity is driven by enzymes already present in the seed and the second phase by the ex novo synthesized lipases. It has been demonstrated that lipase activity can increase in response to elicitors during seed germination. For instance, lanthanide ion (La^+3^) supplemented during soaking of rice seeds and subsequently placed on germination, resulted in significantly higher lipase activity [24]. Likewise, peanut seeds soaked in Cr and Cu solutions at low concentrations enhanced lipase activity. An opposite effect was observed when high concentrations were used [25].

LOX is an enzyme involved in plant physiology, including growth and development, pest resistance, mobilization of storage lipids, and response to wounding and catalyzes deoxygenation of polyunsaturated fatty acids producing conjugated mono hydroperoxides [26]. LOX activity for selenized treatments behaved different when compared to germinated samples, because selenization increased the enzymatic activity by the second day of germination to almost 12,000 UAE/g when the control sample reached only slightly above 6000 UAE/g. During the third day of germination, the selenized treatments reduced the activity, with 2.0 mg/100 g seeds having the highest compared with 0 and 1 mg /100 g seed treatments. After four days germination, the LOX activity decreased by 67% and 55% in sprouts treated with 1 and 2 mg Na_2_SeO_3_/100 g seeds compared to sprouted counterparts without selenium (Figure 1B). This result can be associated with one of the LOX inhibition modes: the reduction of hydroperoxides, essential molecules for the activation of this enzyme [27]. At the same time, the reduction of hydroperoxides may be also associated with the higher OSI observed in oils extracted from selenized sprouts and described in Section 2.1. It has been reported that some selenoenzymes inhibited LOX activity, stabilized lipid structures and diminished lipid hydro peroxides [28], reinforcing the results reported in Table 1 and Figure 1.

### 2.4. Cellular Antioxidant Activity (CAA), β-Carotenoids and Phenolic Compounds of Oils Obtained from Chickpea Seeds and Seeds Germinated with Selenium

**Figure 2:**
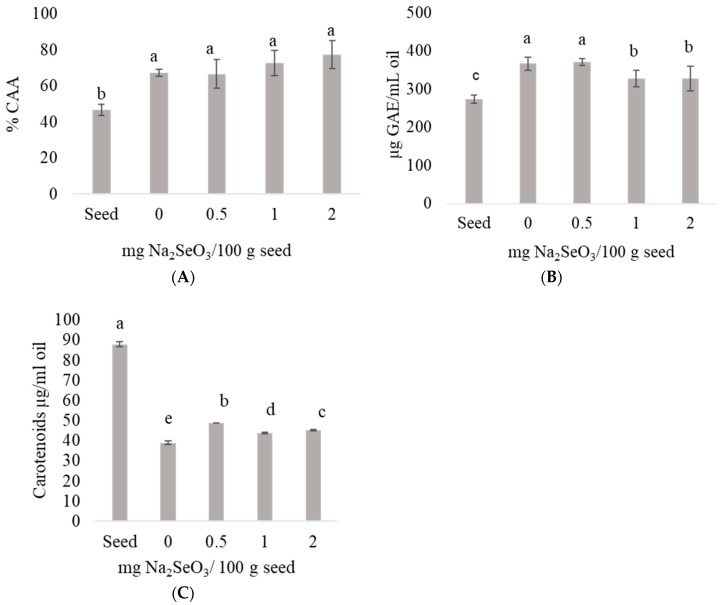
Effect of sodium selenite on cellular antioxidant activity (CAA) (**A**), total phenolic compounds (**B**) and total carotenoids (**C**) measured in chickpea oils obtained from sprouts germinated for four days. Values depicted are averages and bars represent standard deviations. GAE = Gallic acid equivalents. Different letters above bars indicate statistical differences (*p* < 0.05).

The CAA of oil from chickpea sprouts with and without selenium increased from 43% to 66% in comparison to seeds (Figure 2A). No significant differences were found in the CAA of oils extracted from chickpea sprouts treated with and without selenium. In vegetable oils, the CAA is closely associated with antioxidant compounds such as carotenoids and phenolics [29] and the results in this case indicate that the content of phenolic compounds was related to the CAA (r = 0.492) with a *p* = 0.032. Germination enhanced the concentration of these compounds (Figure 2B). Compared to chickpea seed oil, the largest increments of phenolics were 36% and 34% in treatments without selenium and with 0.5 mg Na_2_SeO_3_/100 g seeds, respectively (Figure 2B). The reduction in total phenolic compounds at 1.0 and 2.0 mg Na_2_SeO_3_/100 g seeds agrees with previous reports regarding the dose dependence of phenolics when testing selenium. Besides, there are differences also reported between the effect of Se over the type of phenolic compound [30]. In olive, soybean, sunflower, rapeseed, corn, grapeseed, hemp, flax, rice bran and pumpkin oils, the antioxidant activities greatly depended of the amount of phenolics [31].

The CAA of chickpea oil was inversely associated with the total carotenoid content when a correlation analysis was performed (r = −0.93, *p* < 0.05, Figure 2C). A previous study reported that antioxidant activity of carotenoids is dependent on the concentration and molecular chemistry structure, as well as factors such as oxygen concentration and bioavailability [32]. The total carotenoids decreased significantly in the oil of sprouts germinated for four days. The oil extracted from sprouts germinated without selenium showed 56% less carotenoids compared to the oil from chickpea seeds.

Interestingly, oils extracted from selenized sprouts treated with 0.5, 1.0 and 2.0 mg Na_2_SeO_3_/100 g seeds contained 25%, 13% and 16% higher total carotenoids compared to oils extracted from sprouts germinated without selenium (Figure 2C). The chemical stability of carotenoids is mainly affected by factors such as light, oxygen and free radicals [33]. Likewise, selenium has been widely known for its high antioxidant activity through its action against free radicals and inhibition of lipid oxidation and against production of hydroperoxides. These results show that selenium contained in sprouts exerted a protective effect on the stability of carotenoids.

An interesting negative correlation was observed between total phenolics and carotenoids (r = −0.722, *p* = 0.018) likely related to the influence of selenium in the phenylpropanoid pathway. Among the multiple enzymes of this pathway, phenylalanine ammonia lyase (PAL) is probably the most relevant [34]. According to [2], the PAL activity was enhanced in response to sodium selenite stress in chickpea sprouts resulting in increased concentrations of phenolic compounds.

## 3. Materials and Methods

### 3.1. Production of Chickpea Sprouts and Selenium Determination

The chickpea seeds used herein belonged to the cultivar Blanco Sinaloa (Kabuli type) and were planted and collected in Angostura, Sinaloa, Mexico. The seeds were germinated with different Na_2_SeO_3_ concentrations (0.0, 0.5, 1.0 and 2.0 mg/100 g seeds) during four days at 24 °C and processed as previously described [2]. The total selenium concentration in germinated chickpeas used for this study was previously reported by Guardado-Felix et al [2]. 

### 3.2. Physical and Chemical Properties of Oils Extracted from Chickpea Seeds and Seeds Germinated with Selenium

Oil was extracted and then quantified with the AACC official gravimetric method 03-20.01 [35]. AV and RI were assessed using the standard IUPAC methods 2.201 and 2.102 [36], respectively. OSIs of samples were determined by the AOCS official method Cd 12b-92 [37].

### 3.3. Fatty Acid Profiles (FAMEs) of Oils Obtained from Chickpea Seeds and Seeds Germinated with Selenium

FAMEs were determined using the method described by [38]. The equipment used was: GC- FID (6890N, Agilent Technologies, Santa Clara, CA, USA), equipped with a SP2380 capillary column (30 m × 0.25 mm i.d., 0.20 µm film thickness). Helium was used as carrier gas (1 mL/min). The temperature program was: 50 °C (2 min), ramp to 250 °C, 4 °C/min and 1 min maintenance. Injector and detector temperatures were 260 and 280 °C, respectively.

### 3.4. Lipase and Lipoxygenase (LOX) Activities of Chickpea Seeds and Seeds Germinated with Selenium

Lipase activity was measured by the colorimetric method described by [39]. One unit of lipase activity corresponded to the production of 1 µmol of nitrophenol in 1 min. LOX activity was determined as previously described by [40]. One unit of LOX activity was defined as an increase of 0.1 A234/min.

### 3.5. Cellular Antioxidant Activity (CAA) of Oils Obtained from Chickpea Seeds and Seeds Germinated with Selenium

Oil in water emulsions of chickpea sprouts were prepared using 1% oil in 98% water and 1% Tween 20, homogenized with a microfluidizer (Microfluidics, Newton, MA, USA) at a pressure of 8000 bar. The operation was repeated four times. The CAA of oil chickpea emulsions was evaluated using the protocol described by [41] with slight modifications as follows: CaCo-2 cells were seeded in a 96-well microplate (1 × 104 − 1 × 105 cells/well) and used when reaching their exponential growth phase. After incubation for 24 h, the cell culture medium was discarded and the cells gently washed with 100 μL of PBS (37 °C). Oil emulsions were evaluated at 1:1000 dilutions in cell culture medium with 60 μM dichlorodihydrofluorescein diacetate (DCFH-DA). After 20 min of incubation at 37 °C and 5% of CO_2_, the medium was removed and the cells washed again with 150 μL of warm PBS. Then, 100 μL of PBS with 94.5 μg/mL of 2,2′-azobis(2-amidinopropane) dihydrochloride (AAPH) was added to detect fluorescence (485/538 nm) during 2 h. CAA units were calculated with the following formula: CAA units = 100 − (∫ SA/∫ CA) × 100, where ∫ SA (sample area) is the integrated area under the curve for the sample fluorescence versus time and ∫ CA (control area) is the integrated area from the control curve.

### 3.6. Total Carotenoids of Oil from Chickpea Seeds and Seeds Germinated with Selenium

Carotenoids were isolated from chickpea sprouts oil following the protocol described by [42], using 0.5 mL of chickpea sprouts oil dissolved in 5 mL hexane and vortexed for 30 s with 0.25 mL of 0.5% NaCl. Supernatants were obtained after centrifugation for 10 min at 1500 *g* and measured at 453 nm (Thermo scientific, Madison, WI, USA). Total carotenoids were expressed as β-carotene equivalents.

### 3.7. Total Phenolic Compounds of Oil from Chickpea Seeds and Seeds Germinated with Selenium

Total phenolics of chickpea oil were determined using the Folin–Ciocalteu colorimetric classic method [43]. Absorbances were measured at 765 nm using a microplate reader (BioTek, Inc. Winooski, VT. USA). Total phenolic content was expressed as µg of gallic acid equivalents (GAE)/mL oil.

### 3.8. Statistical Analysis

All measurements were performed in triplicate and results expressed as average ± standard deviations. Data were analyzed by ANOVA procedures and means compared with Tukey’s tests with a significance level of *p* < 0.05 using the Minitab software (V.17.1.0). In the case of CAA, total phenolic and carotenoid Pearson correlations were obtained using the same Minitab software.

## 4. Conclusions

The effect of selenium on the stability and composition of chickpea oil was assessed using different sodium selenite levels (0, 0.5, 1.0 and 2.0 mg/100 g seeds) during germination. When oil from native seeds was compared to 4-day germinated counterparts, the oil content increased from 5.5% to 6.9%, whereas the OSI diminished from 31.8 to 11.4 h. This as a result of dry matter losses during sprouting whereas the observed OSI reduction was associated with enzymatic activity due to the catabolic process of germination. The most abundant fatty acid in all samples was linoleic (57.2 to 59.0%) followed by oleic. The use of selenium improved OSI values up to 46% when the oil extracted from sprouts germinated with 1.0 mg Se/100 g seeds was compared with the counterpart obtained from the germinated control. Lipase activity increased three times after the first day of germination (250 to 1750 UAE/g) for all samples independently of the application of selenium and the highest lipase activity (2000 UAE/g) was observed in sprouts treated with 2 mg of selenite/100 g seeds after the third and fourth germination day. LOX activity, on the other hand, was highest at the end of the programmed germination for non-selenized sprouts. Even though the lipase activity was not affected by selenization, LOX appeared to be associated with the protective effect of glutathione peroxidase (GPX), whose activity is enhanced by selenization. GPX restricts in the first place the amount of hydroperoxides, which in turn reduces LOX activity, increasing the OSI as found in this work. In summary, because LOX activity was lower in selenized treatments and the OSI was higher, the selenium protective effect should be associated with deprivation of hydroperoxides in sprouts. The CAA, on the other hand, increased from around 43% in seeds to 66% for sprouts with and without selenium. The significant difference is attributed mainly to the higher phenolic concentration produced during germination. Selenium exerted a protective effect on the stability of carotenoids. These results suggest that Se-enriched chickpea sprouts could represent an excellent source of oils containing high levels of linoleic acid that possess a high OSI and CAA.

## Figures and Tables

**Table 1:** Physical and chemical properties of chickpea oils obtained from sprouts germinated for four days with different levels of sodium selenite ^1^.

mg Na_2_SeO_3_/100 g Seeds	Oil Content (%)	Refractive Index (RI, 20 °C)	Acid Value (AV, % as Linoleic Acid)	Oxidative Stability Index (OSI, h) ^2^
0.0	6.9 ± 0.19 ^a^	1.468 ± 0.0002 ^a^	12.4 ± 0.06 ^b^	11.4 ± 1.52 ^b^
0.5	6.7 ± 0.02 ^b^	1.467 ± 0.0002 ^b^	15.6 ± 0.24 ^a^	14.6 ± 1.42 ^b^
1.0	6.8 ± 0.01 ^ab^	1.466 ± 0.0002 ^b^	12.3 ± 0.28 ^b^	16.7 ± 3.05 ^b^
2.0	6.8 ± 0.04 ^ab^	1.466 ± 0.0003 ^b^	12.0 ± 0.01 ^b^	13.1 ± 1.90 ^b^
Seed	5.5 ± 0.29 ^c^	1.467 ± 0.0007 ^ab^	5.0 ± 0.44 ^c^	31.8 ± 1.88 ^a^

^1^ Data are expressed as average ± standard deviation on dry weight basis. Values in each column sharing the same letter were not significantly different (*p* < 0.05). ^2^ Oil Stability Index.

**Table 2:** Fatty acid composition (% *w*/*w*) of chickpea oils extracted from sprouts germinated for four days with different levels of sodium selenite.

mg Na_2_SeO_3_/100 g Seeds	Fatty Acids, %			
	Palmitic C16:0	Stearic C18:0	Oleic C18:1	Linoleic C18:2
0.0	8.1	7.8	25.5	58.6
0.5	8.3	7.7	25.0	59.0
1.0	8.3	7.6	25.4	58.7
2.0	8.5	7.7	25.5	58.3
Seed	8.4	7.9	26.5	57.2
